# A Tail-like Assembly at the Portal Vertex in Intact Herpes Simplex Type-1 Virions

**DOI:** 10.1371/journal.ppat.1002961

**Published:** 2012-10-04

**Authors:** Michael F. Schmid, Corey W. Hecksel, Ryan H. Rochat, David Bhella, Wah Chiu, Frazer J. Rixon

**Affiliations:** 1 National Center for Macromolecular Imaging, Verna and Marrs Mclean Department of Biochemistry and Molecular Biology, Baylor College of Medicine, Houston, Texas, United States of America; 2 Department of Molecular Virology and Microbiology, Baylor College of Medicine, Houston, Texas, United States of America; 3 Graduate Program in Structural and Computational Biology and Molecular Biophysics, Baylor College of Medicine, Houston, Texas, United States of America; 4 MRC-University of Glasgow Centre for Virus Research, Glasgow, United Kingdom; Cornell University, United States of America

## Abstract

Herpes viruses are prevalent and well characterized human pathogens. Despite extensive study, much remains to be learned about the structure of the genome packaging and release machinery in the capsids of these large and complex double-stranded DNA viruses. However, such machinery is well characterized in tailed bacteriophage, which share a common evolutionary origin with herpesvirus. In tailed bacteriophage, the genome exits from the virus particle through a portal and is transferred into the host cell by a complex apparatus (i.e. the tail) located at the portal vertex. Here we use electron cryo-tomography of human herpes simplex type-1 (HSV-1) virions to reveal a previously unsuspected feature at the portal vertex, which extends across the HSV-1 tegument layer to form a connection between the capsid and the viral membrane. The location of this assembly suggests that it plays a role in genome release into the nucleus and is also important for virion architecture.

## Author Summary

It is estimated that 90% of the world's adult population is infected with Herpes Simplex Virus (HSV), which is responsible for causing cold sores, blindness, encephalitis, even death. One problem faced by all viruses is how to ensure that their genetic material enters the host cell. In viruses that infect bacteria (bacteriophage), genome transfer from the virus into the cell is carried out by a specialized structure known as the tail. Studies have shown that bacteriophage and herpesviruses share a common evolutionary origin. However, until now, no tail-like structure has been seen in herpes. We have used newly developed electron microscopy based techniques to identify a structure in HSV, which recapitulates features of the bacteriophage tail and may also be involved in release of DNA from the virus particle at the nuclear pore. Such advances in understanding the structures that drive virus replication may suggest new therapeutic targets to counter viral infection.

## Introduction

Human herpesviruses cause a range of diseases from cold sores and chicken pox to congenital defects, blindness, and cancer [1]. Herpes simplex virus type-1 (HSV-1) is a well-characterized virus comprising a ∼200 MDa T = 16 icosahedral capsid containing the viral dsDNA genome, surrounded by a pleomorphic proteinaceous layer (the tegument), and bounded by a glycoprotein-containing lipid envelope [1]. The infectious HSV-1 particle (the virion) varies in size from ∼1550 to >2400 Å, but the capsid has a constant diameter of 1250 Å [2]. The main structural features of the capsid are pentons, which occupy 11 of the 12 capsid vertices; hexons, which form the faces and edges of the icosahedron; and triplexes, which sit between the capsomeres [2]. The twelfth vertex is occupied by a portal [3] that is situated just inside the capsid shell [4]–[6] and is believed to form the route for DNA packaging and release. Techniques for determining structures of icosahedral viruses are well established and have contributed greatly to our understanding of virus biology. Recently, electron cryo-microscopic (cryo-EM) and electron cryo-tomographic (cryo-ET) methods have been developed to allow visualization of non-icosahedral features in such particles. This has allowed the DNA packaging and release machinery (e.g. portal and tail components) of tailed bacteriophages to be studied in detail [7]–[12]. Evolutionary and structural links between tailed bacteriophages and herpesviruses suggest that equivalent machinery might be present in herpesvirus particles. This assumption is reinforced by the structural description of the HSV-1 portal as occupying a similar position and with a similar subunit organization as that of the tailed bacteriophages [3], [6], [13]. However, lack of evidence for a specialized tail-like structure in herpes virions appeared to indicate that the environment provided by the eukaryotic cell rendered such a structure unnecessary. Nevertheless, the delivery of DNA into nuclei by herpesviruses may be considered analogous to the delivery of DNA into bacterial cells by tailed bacteriophages. Although some of the actions performed by the tail (e.g. penetrating the bacterial cell wall) are not germane to herpesviruses, others (e.g. receptor recognition, capsid orientation and signalling) could be required to ensure that the herpesvirus capsid docks and releases its genome at the nuclear pore complex (NPC) [14]. This paper describes a previously unrecognized feature within the tegument of HSV-1 virions, which, by analogy with tailed bacteriophages, may mediate interactions between the portal vertex and cellular components at key points in the viral life cycle.

## Results

### Identifying a unique vertex in virions

**Figure 1:**
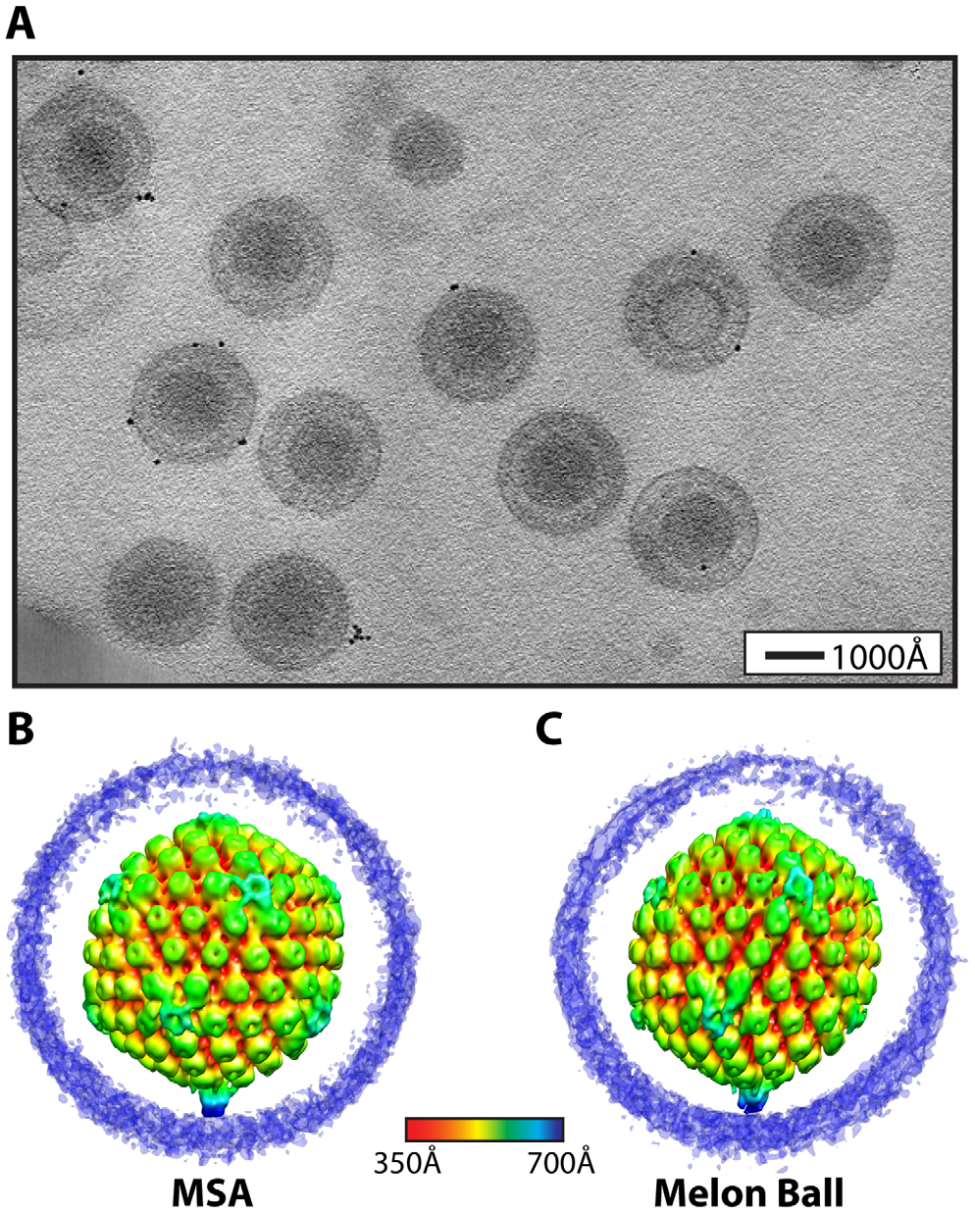
Visualization of a distinctive tegument structure associated with the portal vertex in HSV-1 virions. (**A**) Projection image through a tomogram of HSV-1 virions embedded in vitreous ice (Video S2). (**B–C**) The symmetry-free virion averages generated from 213 subtomograms using MSA-guided classification and melon ball alignment methods, respectively. The maps are shown radially colored, with non-capsid densities trimmed to reveal the underlying capsid. Views are shown looking down a 2-fold axis of symmetry with the portal vertex at the bottom.

Tilt-series comprising consecutive images of ice-embedded HSV-1 virions were collected while the sample was rotated through 120° (Video S1). Tomograms from these tilt series were reconstructed in 3-D (Figure 1A; Video S2) prior to extraction and analysis of subtomograms corresponding to individual virions. We suspected that the presence of any consistently organized tegument at one of the capsid vertices was likely to be obscured by the heterogeneous tegument environment within which it would be located. Therefore, we used vertex-specific search and orientation strategies to look for distinctive features associated with the 12 capsid vertices in each extracted virion subtomogram. To reduce the possibility of introducing bias during data processing, we used two independent computational approaches to locate and extract densities at the 12 vertices. These approaches for symmetry-free alignment were carried out *de novo* and the intermediate models arose from the data itself. The two approaches (described in Materials and Methods), herein referred to as multivariate statistical analysis (MSA)-guided classification and melon ball alignment, both identified a unique vertex, which was used to align each subtomogram before averaging.

**Figure 2:**
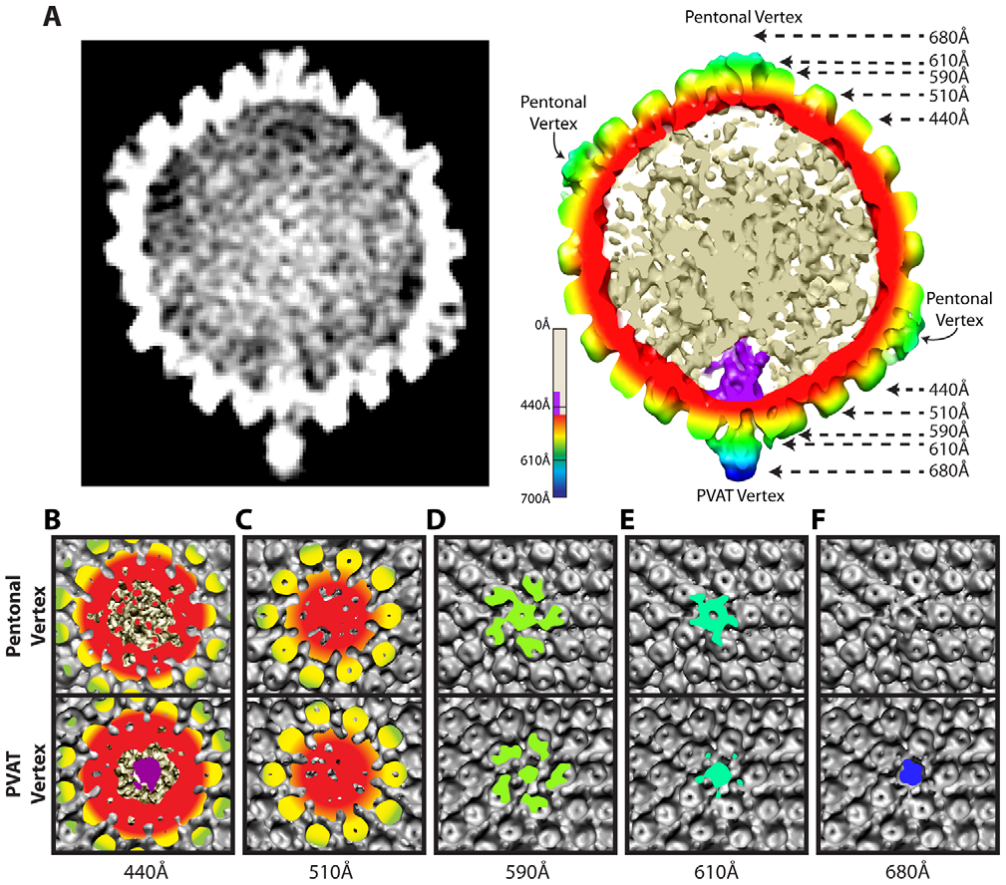
Comparison of portal and pentonal vertices. (**A**) Central section through an MSA-guided virion average. The left-hand panel shows a grey-scale density map. In the right-hand panel, the capsid shell is radially colored, with internal DNA density shown in cream, and portal density in purple. (**B–F**) Individual panels of the capsid shell cut away orthogonal to the axis running through the portal and opposing vertex, at the radii indicated by arrows in (**A**). Views are from the outside of the portal vertex (bottom) and its opposing pentonal vertex (top).

The final averaged density maps of the virion obtained from both methods (Figure 1B & 1C) show the major features of the capsid including pentons, hexons and triplexes. Most of the tegument and envelope density is tenuous, confirming that these components are not consistently arranged from particle to particle [15]. The similarities of the overall capsid structures and dimensions to previous single-particle reconstructions [2] validate our approaches. Eleven of the capsid vertices show densities extending laterally from the tops of the pentons and connecting with the surrounding hexons and triplexes (Figure 1B & 1C), in the same manner as seen in icosahedrally averaged, single particle virion maps [16]. However, both of our symmetry-free reconstructions independently identify a different and unique twelfth vertex that is characterized by a large density extending ∼150 Å from the outer surface of the capsid (Figure 1B & 1C). This feature is shown in a longitudinal section through the virion reconstruction (Figure 2A; Video S3), which also reveals the portal density at the same vertex, protruding into the cavity occupied by the virus genome.

### Organization of the portal vertex

The distinctiveness of the portal vertex is illustrated by comparing equivalent sections through our final model at the portal vertex and the opposing pentonal vertex (Figure 2B–F). The portal occupies a region inside the capsid shell (∼410–490 Å), while the same position under the opposite vertex contains discontinuous densities that presumably represent dsDNA (Figure 2A & 2B). No major differences are seen in the sections containing the capsid floor beneath the vertex (∼510 Å; Figure 2C), suggesting that the presence of the portal does not cause perturbations that are apparent at this resolution. Our virion reconstruction shows density extending outwards from the portal vertex beyond ∼540 Å. This density is roughly cylindrical with a narrow neck that broadens beyond a radius of ∼610 Å (Figure 2D & 2E). We designate this density the portal vertex associated tegument (PVAT). Unlike the pentons, which occupy the other vertices, the PVAT does not have an obvious central channel (Figure 2D & 2E). At the pentonal vertices, densities extend laterally from the top of the penton to the hexons and triplexes [16]. Densities forming a similar pattern are also present at the portal vertex but lie slightly further from the capsid surface, giving the impression of flying buttresses connecting to the middle of the PVAT (Figure 2A & 2E). Discernable density extends much further out from the capsid for the PVAT (beyond 680 Å) than for the pentonal vertices (less than 650 Å; Figure 2A & 2F).

**Figure 3:**
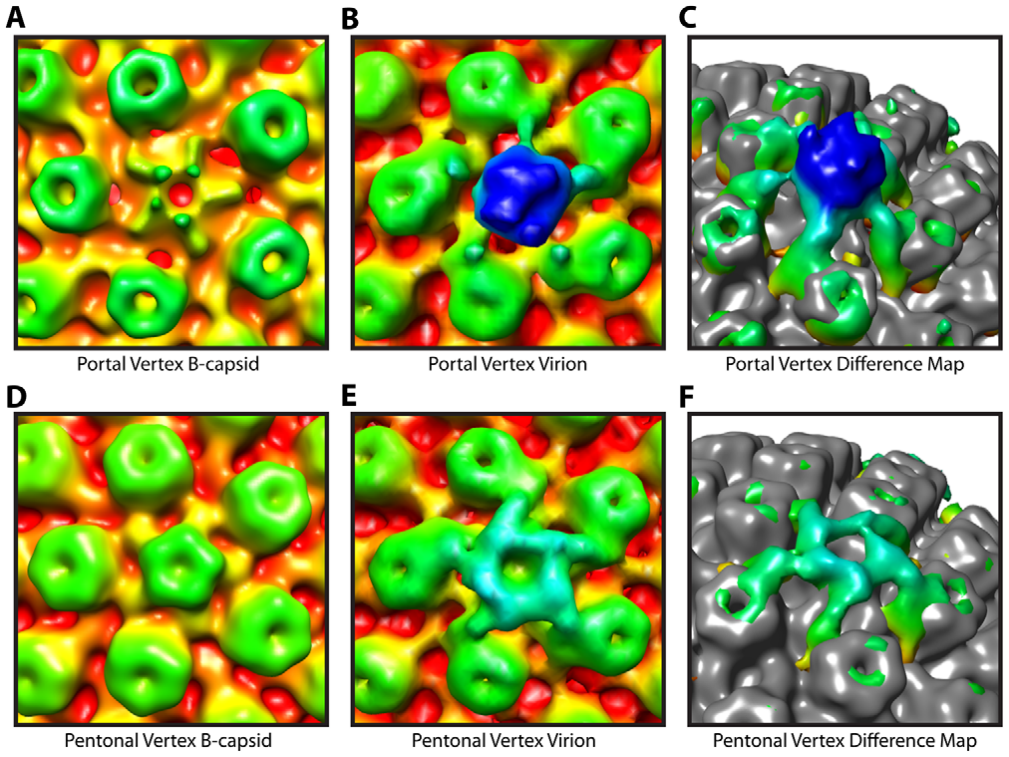
Tegument-capsid interactions at the portal and pentonal vertices. Close up views of portal (**A–C**) and pentonal (**D–F**) vertices. (**A, D**) Symmetry-free B-capsid reconstruction [6] low-pass filtered to 35 Å. (**B, E**) MSA-guided virion average (Figure 1B) shown in the same views as (**A**) and (**D**). (**C, F**) Difference maps (colored) between (**A**) and (**B**), and (**D**) and (**E**) respectively, superimposed on the B-capsid shell (grey), and shown tilted. The weak penton density seen in (**A**) is most likely due to occasional incorrect assignment of the asymmetric orientations [6].

Comparison of our virion map with that of tegument free B-capsids, purified from the nuclei of infected cells, confirm that the PVAT is a tegument feature. Previous studies on B-capsids have shown that significant density at the portal vertex does not extend beyond the floor of the capsid shell at ∼540 Å (Figure 3A) [5], [6]. In contrast, the virion reconstruction shows the PVAT density at the portal vertex (Figure 3B & 3C). Our virion reconstruction also produces the characteristic star-shaped pattern at the pentonal vertices (Figure 3E & 3F) that is seen in an icosahedrally enforced single particle virion reconstruction [16] and which is lacking in a B-capsid reconstruction as described previously (Figure 3D) [6].

### Capsid envelope spacing

**Figure 4:**
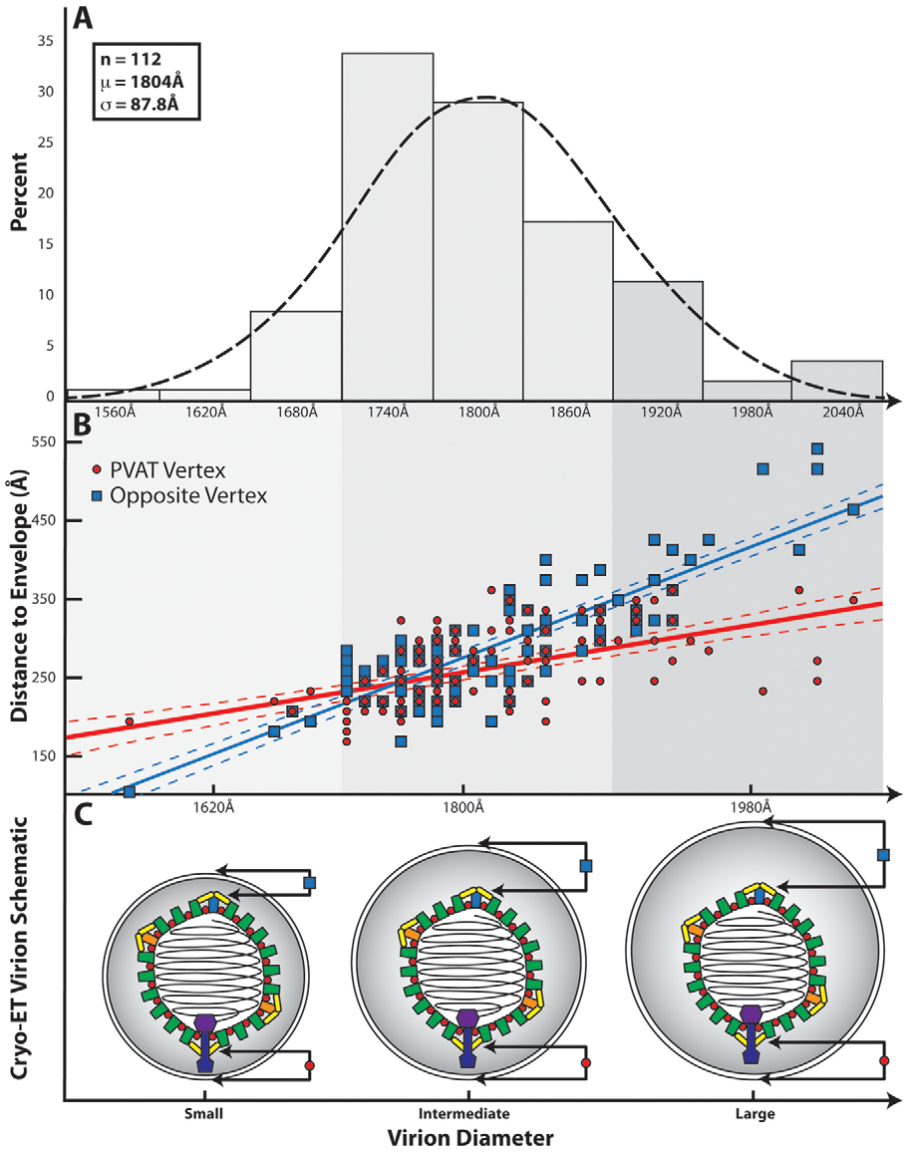
Influence of the PVAT on capsid-envelope eccentricity. (**A**) Distribution of HSV-1 virion diameters for 112 of the virions used in the final reconstruction. (**B**) Graph showing the separation of the envelope from the capsid shell (defined as 625 Å from the center of the capsid) measured at the portal vertex (red line) and at the opposing pentonal vertex (blue line), and plotted as a function of virion diameter. The difference in slope between the two lines is a clear indication that the capsid is not randomly positioned within the envelope. Note that the vertex closest to the envelope is not always the portal vertex. (**C**) Schematic illustrating the spatial relationship between the HSV-1 capsid and the envelope for small, intermediate, and large sized virions, as predicted from (**B**).

A previous tomographic study on purified virions showed that the capsid is asymmetrically placed within the envelope [15]. To investigate this, the capsid-to-envelope separation for each virion was measured at the portal and opposing pentonal vertices. These values were plotted as a function of virion diameter, which varies among particles (Figure 4A & 4B). The resulting graph shows two separate distributions, with the slope of the portal vertex measurements being less steep than that for the opposite vertex (p-value<0.001). This indicates that the portal-to-envelope separation is more consistent than that at the opposite vertex, regardless of the overall size of the envelope (Figure 4C).

## Discussion

The detection of the PVAT is technically difficult because its presence at only one vertex makes it unsuitable for icosahedral reconstruction. Furthermore, it is surrounded by inconsistently arranged tegument material of roughly similar density and so it differs from a bacteriophage tail, which is not obscured by other structural components. We were able to visualize it here because the methods used were sufficiently sensitive to distinguish the set of unique signals provided by the portal vertex and use this information to direct the alignment of each subtomogram. Aligning them based on the density distribution at the portal vertex meant that the PVAT density was reinforced in the final reconstruction, while that of the surrounding pleomorphic tegument, was weakened. The MSA-guided and melon ball alignment procedures produced the same particle orientation for 151 of the 214 particles. Analysis of the other 63 particles suggested that the majority had been reliably assigned by MSA-guided alignment whereas melon ball alignment was more frequently erroneous due to systematic bias from aligning to the missing wedge. It should be emphasized that in the MSA-guided method, the assignment of the portal vertex is made on a per-particle basis. In contrast, the all-vs-all melon ball alignment simply compares all the vertices in one particle to all the vertices of another particle without any starting assumption about any of the vertices. Because the two independent and unrelated alignment methods generated the same final structure, we attach high confidence to the resulting description of the PVAT.

It is likely that the PVAT does not constitute a self-contained structure but represents a set of proteins that are consistently arranged with respect to the portal vertex and are continuous with the heterogeneous surrounding tegument. In this sense, it resembles the star-shaped densities seen at the tops of pentons, where the surface displayed in the reconstruction delineates a boundary between the constant and changeable parts of the tegument.

The PVAT occupies a position that would enable it to carry out several of the receptor binding and signalling functions that in tailed bacteriophages are performed by the tail. In these bacteriophages, the tail plays a key role in docking the virus to its host and controlling entry of the virus genome into the host cell. In herpesvirus-infected cells, the PVAT may play an equivalent role in docking the capsid to the NPC as well as in signalling the proper conditions for release of the viral DNA. Ensuring that the viral genome is released only when the capsid reaches the nucleus would require interaction between components of the virus particle and the NPC [17].

The composition of the PVAT cannot be directly determined from our reconstructions. The likelihood that it is DNA leaking from the capsid is small, as all existing evidence suggests that the viral genome is fully retained within capsids until its release is triggered at the nuclear pore. Furthermore, the size and shape of the PVAT are consistent with it being a complex of proteins but not with it being formed by a single DNA strand. Two proteins that have been shown to have roles in NPC recognition [18], [19] and DNA release [20], [21], and are therefore likely to be present at the portal vertex, are pUL25 and pUL36. pUL25 together with a second protein, pUL17, has been identified as forming the capsid-vertex-specific component (CVSC) on capsids purified from infected cell nuclei [22]–[25]. The CVSC occupies a position overlying triplexes Ta and Tc (as designated in [26]); adjacent to, but not extending to, the pentons. This position corresponds to that of the distal portions of the star shaped densities that are seen in our map connecting the pentons to the peripentonal triplexes and hexons (Figure 3E). The proximal portions of our star shaped densities that are in contact with the pentons, are also present in virions that have been stripped of their envelopes and much of their tegument where they have been shown to be formed by pUL36 [27]. The common geometry displayed by the outer portions of the star-shaped densities at the pentonal vertices (Figure 3E, 3F) and the flying buttresses at the portal vertex (Figure 3B, 3C) supports a hypothesis that they are formed by these same proteins. Interestingly, the star-shaped density seen at the pentonal vertices can only account for a fraction of the mass of pUL36 (∼336 kDa) [16], implying that much of it is unresolved at these locations. Therefore, reorganization of pUL36 induced by the unique environment at the portal vertex could allow it to contribute to the distinctive PVAT structure.

An interesting aspect of the PVAT is its potential role in orchestrating the arrangement of tegument and envelope around the capsid. In many of the individual virions, the distal end of the PVAT was found to lie close to the envelope. This observation, combined with the relatively fixed separation of the portal vertex and envelope evident from Figure 4B, appears to indicate that the PVAT is acting in the manner of a tether, connecting the envelope to the capsid (Figure 4C). Such behavior would suggest that formation of the virion envelope, which takes place by budding into golgi-derived vesicles, could be influenced by the orientation of the capsid around the portal vertex.

This study demonstrates the increasing power of cryo-ET and 3-D image processing techniques to uncover unexpected features, even in a virus as extensively studied as HSV-1, and illustrates the potential of such approaches to enhance our understanding of other important molecular machines.

## Materials and Methods

### Data acquisition

Herpesvirus virions grown in BHK-21 cells were prepared by banding on Ficoll gradients [28], mixed with 100 Å gold particles (British Biocel) and vitrified. Tilt series were collected in SerialEM [29] on a JEM2200 FS electron microscope equipped with a Gatan 914 cryo-stage (Video S1). Energy filtered images were recorded, with a slit width of 30 eV, on a Gatan Ultrascan 4k×4k CCD camera at a nominal magnification of 20,000× (detector magnification of 28,195×), corresponding to a pixel size of ∼5.3 Å/pixel, which was binned by a factor of two to ∼10.6 Å/pixel. The intended tilt range was −60° to +60° in 2° intervals (intended defocus of ∼4 µm). IMOD [30] was used to produce tomographic reconstructions. 240 intact virions were extracted as subtomograms from 11 tilt series.

### Image processing

**Figure 5:**
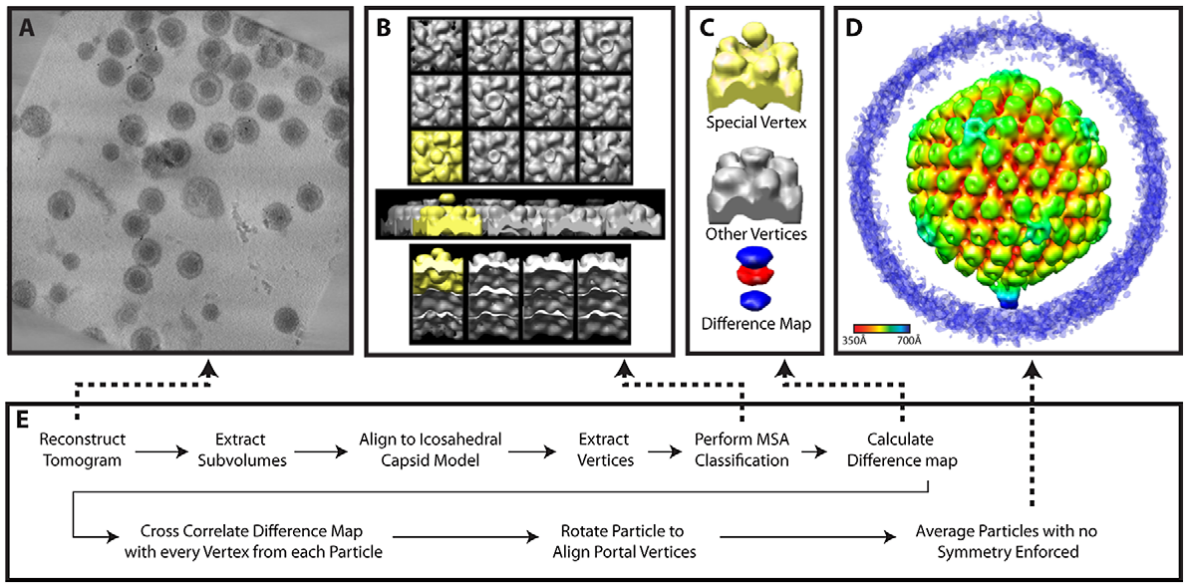
Workflow for MSA-guided classification. (**A**) Projected slices from a representative tomogram of HSV-1 virions. (**B**) 12 MSA-derived class averages, top, side and lower views. The class average representing a putative portal vertex is shown in yellow. (**C**) The putative portal vertex (top), the average of all other vertices (middle) and the difference map calculated from them (bottom), with the positive difference densities in blue, and negative difference density in red. (**D**) Final MSA-guided average, radially colored. (**E**) Flow chart of the procedure (described in detail within the Materials and Methods).

**Figure 6:**
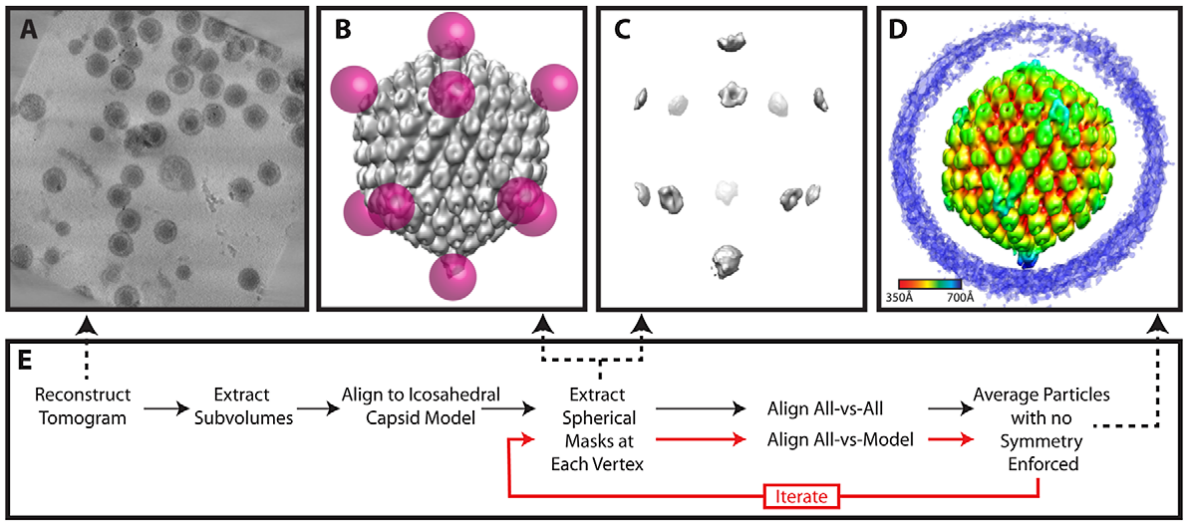
Workflow for melon ball alignment. (**A**) Projected slices from a representative tomogram of HSV-1 virions. (**B**) 12 spherical masks (magenta), in an icosahedral arrangement, superimposed on a map of the capsid (grey), showing the relationship of the masks to the capsid surface. (**C**) Density extracted by the masking procedure in (**B**). After generating an initial model, the model may be updated iteratively by realigning the particles (red lines). (**D**) Final melon ball aligned average, radially colored. (**E**) Flow chart of the procedure (described in detail within the Materials and Methods).

For both MSA-guided classification (Figure 5) and melon ball alignment (Figure 6) virion subtomograms were aligned to an icosahedral model derived from a single-particle reconstruction of the virion [16]. This alignment was carried out in the icosahedral asymmetric unit of the capsid and produced particles oriented according to a standard icosahedral setting [31], which took no account of any special vertex at this stage. To assess quality, icosahedral symmetry was imposed. If the hexon structure was not visible, one attempt was made to realign the particle. About 85% of the particles were successfully oriented as judged by this criterion.

### MSA-guided classification

#### Multivariate Statistical Analysis (MSA)

A least squares line through a two dimensional set of points describes the major trend of variation among those points. This line, also referred to as an eigenvector, describes the direction of variability in the data. The magnitude of this vector (i.e. its eigenvalue) denotes how much of the variability in the data is accounted for by this one-dimensional line. In a similar fashion, sets of 3-D volumes can also be so classified, where the dimensionality of the analysis is equal to the number of voxels per volume [32], [33]. As such, MSA performed on these volumes produces numerous eigenvectors, the largest of which express the majority of the variability in the data. This variability can be visualized in 3-D by a series of individual volumes (i.e. eigenimages) that characterize each eigenvector. 3-D averages (i.e. class averages) can be created from images clustered according to these major eigenvectors. Image processing methods employing these principles are often referred to as MSA.

#### Application of MSA to HSV-1 virion data

The initial stage of our MSA-guided classification used 76 particles from 4 tilt series aligned to an icosahedral model [16]. The 12 vertices from each unsymmetrized particle (i.e. raw subvolume) were computationally extracted and 5-fold symmetry was applied. These subvolumes (420×420×340 Å) were centered at 540 Å from the center of the particle, corresponding to the volumes shown in the class averages (Figure 5B). Thus they extend from within the capsid shell to a position beyond the top of the penton and would encompass the penton, portal, peripentonal hexons, and parts of the tegument. These 912 subvolumes were subjected to MSA.

We observed that the eigenimages from the highest 3 eigenvalues looked similar to the vertices themselves (even at the surrounding hexons). This argued for a global, non-structural cause for the variability expressed in these eigenvalues [34]. When we used these 3 eigenvectors for classification, the vertices were clearly classified based on missing wedge effects, as evidenced in the class average maps. We therefore chose the next 7 highest eigenvectors, and requested 12 classes (Figure 5B). While eleven of the class averages resembled each other (shown in grey), one (shown in yellow) had extra density extending outward from the vertex, additional extra density inside the particle, and lower density at the position where the other classes had pentons. The external density was truncated at the top because we wanted to exclude density that could have been derived from the envelope.

We created a difference map by subtracting the average of all vertices from this putative portal vertex (Figure 5C). The difference map showed positive difference density (blue) both outside and inside the capsid, and negative difference density (red) at the position that is occupied by a penton at other vertices. No other differences were seen. This difference map “signature” was cross-correlated with all the vertices in each particle to redetermine the location of the portal vertex, both in the original set of particles and in a new group of 198 particles from 7 additional tilt series that became available at this stage. For each particle, the vertex with the highest correlation was identified. We found that for 65 of the original 76 particles and 149 of the additional 198 particles, that vertex had a correlation value 1.5 standard deviations above the average for all 12 vertices. The particle maps were rotated to bring the putative portal vertices into alignment. This led to a single average for all particles (Figure 1B; Figure 5D) (accession code: EMD-5452).

### Melon ball alignment

For melon ball alignment, 12 spherical masks were applied to the best 65 icosahedrally aligned but unsymmetrized particles selected by MSA. Each mask was 700 Å from the center of the particle and had a radius of 110 Å, so that only density corresponding to the distal parts of penton and nearby tegument remained (but not the rest of the capsid shell or portal) (Figure 6B & 6C). Masking the particles in this way ensured that subsequent alignments only paid attention to density at the vertices and disregarded the rest of the particle. The 65 volumes were binned by 2 and low pass filtered to 40 Å. All-vs-all cross-correlation alignment [35] was performed at the 60 possible icosahedral orientations. We applied the rotation that yielded the highest cross-correlation to the original particles to produce an average. This average was melon ball masked and used for realigning these 65 particles and the additional 149 particles as selected by MSA, producing the final averaged map (Figure 1C; Figure 6D) (accession code: EMD-5453).

### The missing wedge

The alignment procedure we used, including the precautions taken to minimize the effect of the missing wedge in single particle tomographic alignment and averaging, has been described in detail [35]–[37]. Correctly accounting for the missing wedge is an essential part of 3-D tomographic alignment and averaging, and has also been implemented by others [38]. Briefly, the cross-correlation maps generated for each rotational search location are normalized through this process, which has the effect of equalizing the total power, in Fourier space, of the complex product of the two particles. Accordingly, it is possible to compensate for the varying number of “zero” terms generated as the missing wedges of the two maps interact with each other and the “non-zero” data. In this way, alignment will not be influenced by falsely high correlation scores due to alignment of the missing wedges.

### Statistical analysis of the capsid-envelope separation

Of the 214 virions used in the final symmetry-free reconstruction, 112 had well visualized envelopes at both the portal and opposite vertices. For Figure 4, statistical analyses of the capsid-envelope separation were performed using SAS software Version 9.2 (SAS Institute Inc., Cary, NC, USA). Virion diameter was approximated by summing the separation from the envelope to the capsid shell for both pentonal and portal vertices together with a constant of 1250 Å (the diameter of the capsid shell). These separations were modelled as a function of virion diameter using PROC GLM (generalized linear model).

## Supporting Information

Video S1 Tilt series of vitrified HSV-1 virions.
(M4V)

Video S2 Tomogram reconstructed from the tilt series shown in Video S1.
(M4V)

Video S3 Video showing a 3-D reconstruction of the HSV-1 virion.
(MOV)
